# Serum Vitamin D_3_ Concentration, Sleep, and Cognitive Impairment among Older Adults in China

**DOI:** 10.3390/nu15194192

**Published:** 2023-09-28

**Authors:** Yuning Xie, Chen Bai, Qiushi Feng, Danan Gu

**Affiliations:** 1 School of Labor and Human Resources, Renmin University of China, Beijing 100872, China; xieyuning1998@ruc.edu.cn (Y.X.); baichen_1023@126.com (C.B.); 2 Centre for Family and Population Research, Department of Sociology and Anthropology, National University of Singapore, Singapore 119077, Singapore; 3 Independent Researcher, Nanjing 210042, China

**Keywords:** serum 25-hydroxyvitamin D_3_ (25(OH)D_3_), sleep quality, sleep duration, cognitive impairment, older adults, oldest-old, blue zones, longevity areas, China

## Abstract

Background: Cognitive decline in older adults has become one of the critical challenges to global health. This study aims to examine both cross-sectional and longitudinal associations of levels of serum 25-hydroxyvitamin D_3_ (25(OH)D_3_) (briefed as VD3) concentration and sleep quality/duration, especially their interactions, with risk of cognitive impairment among older adults in China. Methods: We utilized a special subsample of adults aged 65–105 years (individuals = 3412, observations = 4816) from eight provinces in China derived from the 2011/2012 and 2014 waves of the Chinese Longitudinal Healthy Longevity Survey. Cognitive impairment was measured by the Mini-Mental State Examination scale. Sleep quality was classified as good versus fair/poor, and sleep duration was classified into short (<7 h), normal (≥7 but <9 h), and long (≥9 h). The VD3 concentration was divided into three levels: deficiency (VD3 < 25 nmol/L), insufficiency (25 nmol/L ≤ VD3 < 50 nmol/L), and sufficiency (VD3 ≥ 50 nmol/L). A wide set of covariates that include demographics, socioeconomic status, family support, health practice, and health conditions was adjusted for robust findings. Multilevel random intercept logit regression models were used to examine the cross-sectional associations between VD3, sleep, and cognitive impairment, whereas logit regression models were applied to investigate the longitudinal associations. Results: In the cross-sectional analyses, when all covariates were adjusted, VD3 sufficiency was significantly associated with a 33% lower risk of cognitive impairment compared with VD3 deficiency; good sleep quality was associated with 34% lower odds of cognitive impairment compared with fair/poor sleep quality; sleep hours were not associated with cognitive impairment, although a long sleep duration (≥9 h) was associated with 30% higher odds of being cognitively impaired when baseline health was not controlled. Interaction analyses reveal that VD3 sufficiency could help to additionally reduce the risk of cognitive impairment for good sleep quality and normal sleep hours. In the longitudinal analyses, the association of VD3 sufficiency remains significant, whereas sleep quality and sleep duration were not significant associates. Conclusions: Good sleep quality, normal sleep hours, and VD_3_ sufficiency are positively associated with good cognitive function. VD3 sufficiency could enhance the associations between sleep and cognitive impairment.

## 1. Introduction

With the rapid aging of the global population, cognitive impairment has become a critical issue with far-reaching implications for healthcare, economies, families, and societies [1]. The challenge is likely more pressing in mainland China (hereafter China) because of its unprecedented population aging process [2] and relatively high prevalence of cognitive impairment among older adults [3,4]. Although cognitive impairment, especially severe impairment or dementia, has recently become a global public health challenge, effective medical treatments are mostly limited or absent [5,6,7]. Accordingly, early identification of modifiable risk factors that impact age-related cognitive impairment is vital for implementing appropriate preventive interventions, which is essential to prevent or at least delay the progression of developing dementia [8].

Plenty of literature has demonstrated that sleep disorders are significantly relevant to the risk of cognitive impairment with aging [9,10,11,12,13,14,15,16]. The findings are generally consistent with the majority of cross-sectional studies suggesting that a short sleep duration (usually less than 7 h) [17,18,19], a long sleep duration (usually 9 h or more) [20,21], or both (i.e., a U-shaped relationship or a V-shaped relationship) [22,23,24,25] are linked to an increased risk of cognitive impairment. Some studies have also suggested that poor sleep quality may be linked to an increased risk of cognitive impairment as well. However, a small number of studies have observed no association between sleep hours/quality and cognitive decline [26]. Some longitudinal studies investigate the association between short (≤6 h per day) or long (≥9 h per day) sleep durations at baseline and cognitive decline at follow-up. However, the results were also mixed, with some reporting a positive association, whereas others reported no association [10,27,28]. More recently, the roles of interactions between some biomarkers (tau/amyloid-β proteins) [29] or psychological factors (depression) [30] and sleep patterns in association with cognitive function have been explored, which has helped to better understand how the relationship between sleep and cognitive function could be affected by other factors.

The level of serum 25-hydroxyvitamin D_3_ (25(OH)D_3,_ briefed as VD3) concentration is a steroid hormone with crucial roles in the regulation of calcium homeostasis and bone metabolism [31]. VD3 deficiency is increasingly regarded as a public health concern worldwide. Most adverse health outcomes induced by VD3 deficiency are skeletal-related, such as rickets, osteoporosis, and osteomalacia [31,32]. However, in recent decades, a growing body of research has indicated that VD3 can have much more wide-ranging anti-inflammatory and immune-modulating effects on human extra-skeletal systems [33,34]. A high level of VD3 concentration has been proven to be neuroprotective by influencing neurogenesis, the expression of neurotrophic factors, detoxification, and phagocytosis of amyloid-beta plaques [35]. For instance, a study by Llewellyn et al. found an association between low VD3 levels (<10 ng/mL or 25 nmol/L) and an increased risk of cognitive decline in a group of 858 community-dwelling older participants in Italy [36]. Similarly, Da Rosa et al. demonstrated that VD3 deficiency was associated with higher prevalence of cognitive decline among the oldest-old population aged 80 years or older in Brazil [37].

The relationship of VD3 deficiency with sleep among older adults has also been explored. Plenty of literature has found that low levels of VD3 are related to short or long sleep durations, decreased sleep efficiency, and sleep fragmentation [38,39,40,41,42,43,44]. For instance, based on 207 women aged 55 years or older in Macao, China, one study found that both short and long sleep durations were significantly associated with VD3 deficiency [40]. Using a larger sample size with 1614 Korean adults aged 60 to 80 years, another study found that a short sleep duration was associated with a lower level of VD3 [41]. Yet some other studies have found that the association between lower levels of VD3 and sleep disorder was largely explained by potential confounders [45]. More importantly, how sleep and the level of VD3 concentration interplay in their associations with cognitive impairment among older adults remains open to investigation. One limitation of the existing literature is the lack of empirical evidence on the relationship between VD3 levels, sleep, and cognitive impairment from the longitudinal perspective. Most studies in the existing literature have been cross-sectional, with only a few exceptions [46]. Another limitation is the lack of representation of the oldest-old population in the study samples, which is crucial for studying healthy longevity.

This study addresses these limitations by conducting a community-based investigation in eight areas of China with a relatively high proportion of longevous individuals. The study aims to examine the cross-sectional and longitudinal associations between the level of VD3 concentration, sleep quality, sleep duration, and cognitive impairment, as well as the interaction between the level of VD3 concentration and sleep quality/duration in relation to cognitive impairment among older individuals.

## 2. Materials and Methods

### 2.1. Study Population

The data were from a subsample of the Chinese Longitudinal Healthy Longevity Study (CLHLS). The CLHLS was a community-based face-to-face nationally representative survey focusing on the health and well-being of older adults in China [47,48]. By 2023, it has conducted 9 waves in 1998, 2000, 2002, 2005, 2008–2009 (briefed as 2008), 2011–2012 (briefed as 2011), 2014, 2017–2019 (briefed as 2018), and 2021, respectively, covering 22 of 31 provinces in China.

In 2008, 2011, 2014, and 2018 waves, the CLHLS conducted a special sub-survey called the Healthy Aging and Biomarkers Cohort Study (HABCS) or the biomarker sub-study in eight areas/sites to collect data on plasma biochemistry examination (GLU, BUN, TG, UA, etc.) and the complete blood count (CBC) [49]. The purpose of this HABCS is to enhance the research on the role of the interactions between biomarkers/genetic and environmental factors in determining health and longevity [47,48,49,50]. These eight areas are from eight provinces, each of which has a relatively higher proportion of long-lived people (aged 90 or older) than other areas in China. The areas are known as longevity areas in China (or internationally known as blue zones) and are recognized by the China Association of Gerontology and Geriatrics (CAGG) (http://www.cagg.org.cn/portal/list/index/id/30.html, accessed on 25 September 2023) [50,51].

Our current research used the 2011 and 2014 waves of HABCS due to the unavailability of VD3 in the 2008 wave and the lack of public accessibility to the 2018 wave thus far. A total of 3412 older adults aged 65 years or older in these eight areas were sampled in the 2011 and 2014 waves. Out of 2354 older adults who were interviewed in the 2011 wave, 1404 older adults were re-interviewed in the 2014 wave. In other words, this study included 4816 observations from 3412 individuals. The dataset used in this study is publicly available at the National Archive of Computerized Data on Aging, University of Michigan (https://www.icpsr.umich.edu/web/NACDA/studies/37226, accessed on 25 September 2023).

### 2.2. MMSE and Cognitive Impairment

The Chinese version of the Mini-Mental State Examination (MMSE) is adapted from Folstein’s original scale to be more practical for older adults and patients with dementia in China, where the literacy level is relatively low [47,52]. The Chinese version of MMSE is composed of 23 questions from six domains (orientation, registration, attention and calculation, copy and drawing, recalling, and language) with a range of scores 0–30 [47]. The data quality of this indicator is relatively high [52]. Unlike objective questions that can be answered by informants, all MMSE questions have to be answered by the sampled older adults themselves. As a result, about 12% of the respondents were “not able to answer” these MMSE questions, mostly due to cognitive, mental, or physical reasons. We recoded these responses as “wrong” if they were due to cognitive or mental health illnesses [50]. For the remaining cases, we followed a common practice in the field to impute them based on multivariable regression models [53,54,55]. The imputation assumes that answers for MMSE questions would be the same for older persons with missing values as those who did not have missing values on MMSE questions if the former group has the same demographics, socioeconomic status, family/social support, health behaviors, and physical health conditions as the latter group. Following the original criterion, participants with an MMSE score below 24 were considered cognitively impaired, while those with scores of 24 or higher were considered cognitively unimpaired [47,52].

### 2.3. VD3 Measurement

In HABCS, a fasting venous blood sample was collected in heparin anticoagulant vacuum tubes and centrifuged at 20 °C, 2500 rpm for 10 min. The plasma was isolated, frozen at −20 °C, shipped on wet ice to the central laboratory at Capital Medical University in Beijing, and then stored at −80 °C until analysis [56]. The level of VD3 was measured using an enzyme-linked immunoassay by Immunodiagnostic Systems Limited (IDS Ltd., Boldon, UK). The inter-assay and intra-assay coefficients of variation were <10% and <8%, respectively [57]. Following the established practice in the literature [51], the level of VD3 concentration was classified into VD3 deficiency (25(OH)D_3_ < 25 nmol/L, reference), VD3 insufficiency (25 nmol/L ≤ 25(OH)D_3_ < 50 nmol/L), and VD3 sufficiency (25(OH)D_3_ ≥ 50 nmol/L).

### 2.4. Sleep Quality and Duration

A self-reported question, “How do you rate your overall sleep quality recently?” was used to assess the subjective sleep quality in HABCS, which is similar to the 6th question in the Pittsburgh Sleep Quality Index (PSQI) with good reliability and validity [53,54,55,58]. The five categories of answers were dichotomized into very good/good versus fair/bad/very bad. The sleep duration was measured by “How long do you typically sleep every day, including napping?” For the sake of simplicity, as carried out by other studies [40], we classified the sleep duration into three groups: short duration (≤6 h or <7 h), normal duration (≥7 and <9 h, or 7–8 h), and long duration (≥9 h). Other categorizations were also tested [30,59], and the results were more or less similar.

### 2.5. Potential Confounding Variables

To ensure the robustness of our findings, a wide set of covariates were adjusted for more robust results, including demographic characteristics, socioeconomic status (SES), family and social connections, health practices, and health conditions. These covariates have been evidenced to be associated with sleeping, mortality, nutrition, and levels of VD3 concentration [28,37,53,54,55,60]. Demographic variables were composed of chronological age, sex (men vs. women), ethnicity (Han ethnicity vs. non-Han minority), and residence (urban vs. rural). Socioeconomic status was proxied by years of schooling (0, 1–6, and 7+), primary lifetime occupation (professional job vs. others), economic independence (yes vs. no), being able to make ends meet (yes vs. no), family economic condition (rich vs. fair/poor), and adequate access to health services (yes vs. no). Family and social connections included marital status (currently married vs. not married) and co-residence status (with household member(s) vs. living alone/institutionalized). Health practices consisted of current smoking (yes vs. no), current drinking (yes vs. no), and regular exercise (yes vs. no). Health conditions were measured by disabilities in activities of daily living (ADL, yes vs. no), instrumental ADL (IADL, yes vs. no), having 1+ chronic condition (yes vs. no), and self-reported poor health (yes vs. no).

### 2.6. Analytical Strategies

Nested multilevel logistic regression models with random intercept were used to examine the cross-sectional and longitudinal associations between the level of VD3 concentration, sleep quality/duration, and cognitive impairment. The application of the multilevel random intercept models is to adjust for intrapersonal correlation since some participants have two observations in the 2011 and 2014 waves. The purpose of the nest model design is to better capture how the relationships between the levels of VD3 concentration, sleep quality/duration, and cognitive impairment were modified by different covariates. Such design is common in literature [20,23,25,53,54,55].

Model I adjusted demographic characteristics, years of schooling, a geographic variable reflecting the eight areas, and the year of waves. The inclusion of the geographic variable and the survey year is to adjust for potential non-random sampling errors in these eight areas and two waves. Model II additionally incorporated other socioeconomic status (economic status, primary occupation, and access to medical services), family and social connections, and health practices. Model III further adjusted health conditions. The interaction effects between the levels of VD3 and sleep quality/duration on cognitive impairment were tested in Model IV by adding an interaction term between the level of VD3 concentration and sleep quality and in Model V by adding an interaction term between the level of VD3 concentration and sleep duration. For better clarity, a table summarizing the model design is presented in Supplementary Materials (see Table S1).

For longitudinal associations between VD3, sleep quality, sleep duration, and cognitive impairment, a similar approach was applied among 1404 participants who were interviewed in both the 2011 and 2014 waves. The outcome of the longitudinal analysis is the cognitive function in 2014, whereas all other variables used their information in 2011. Sensitivity analyses were additionally conducted for both cross-sectional and longitudinal analyses using multiple linear regressions, and the conclusions are the same (see Tables S2 and S3 in Supplementary Materials). All statistical analyses were executed using STATA 18.0.

## 3. Results

Table 1 presents the unweighted descriptive statistics of the sample for studying variables. The mean value of the level of VD3 concentration of all samples was 42.6 nmol/L (SD = 22.2), with about 20% being VD3 deficient, 50% insufficient, and 30% sufficient. Comparing the sample characteristics across the three levels of VD3 concentration, those VD3 deficient tended to be older, women, currently unmarried, co-resident with family, non-smokers, non-alcohol drinkers, and cognitively impaired. They also had a lower SES, higher ADL and IADL disability, and longer sleep hours. These VD3 deficient samples were more likely recruited from the survey site of Shandong Province.

Table 2 reports the cross-sectional associations between the level of VD3 concentration, sleep quality, and impaired cognition from the pooled cross-sectional data. When controlling for demographic factors, years of schooling, provinces of residence, and survey years, the insufficient and the sufficient VD3 concentration were associated with 43% (odds ratio (OR) = 0.57, *p* < 0.001) and 63% (OR = 0.37, *p* < 0.001) lower odds of being cognitively impaired, respectively, compared to VD3 deficiency (Model I). Good sleep quality was associated with 51% (OR = 0.49, *p* < 0.001) lower odds of being cognitively impaired than fair/poor sleep quality. The long sleep duration (≥9 h) was associated with 30% higher odds (OR = 1.30, *p* < 0.05) of being cognitively impaired compared to the normal sleep duration (7–8 h).

When economic conditions, primary lifetime occupation, access to adequate medical services, family and social connections, and health practices were additionally adjusted, these lower odds only changed slightly (Model II). When health conditions were included in the analysis (Model III), the ORs for the VD3 insufficiency and the long sleep duration were no longer statistically significant. However, the ORs for vitamin D3 sufficiency and good sleep quality remained statistically significant, albeit reduced to 33% and 34%, respectively.

Regarding the interactions between sleep quality and VD3 concentration level (Model IV), it was found that participants with good sleep quality and sufficient VD3 concentration had 47% lower odds (OR = 0.53, *p* < 0.01) of cognitive impairment compared to those with fair/poor sleep quality and deficient VD3 concentration. This suggests that the presence of both good sleep quality and sufficient VD3 concentration enhanced the associations between sleep quality and VD3 concentration in relation to cognitive impairment. However, no significant benefit was observed for VD3 sufficiency in reducing the odds of cognitive impairment for participants with fair/poor sleep quality.

In the case of the interactions between sleep duration and VD3 (Model V), both VD3 insufficiency and VD3 sufficiency were associated with a lower likelihood of cognitive impairment within each of the three sleep durations, but the reduction in odds seems more notable for the short and normal sleep durations than for the long sleep duration.

In the longitudinal analyses (Table 3), compared to VD3 deficiency, VD3 insufficiency and sufficiency were associated with 37% (OR = 0.63, *p* < 0.05) and 55% (OR = 0.55. *p* < 0.01) lower odds of being cognitively impaired, respectively, when all covariates were controlled for (Model III), which were slightly lower than those reduced odds in Models I and II when only a subset of covariates were adjusted for. Sleep quality and sleep duration were not significantly associated with cognitive impairment. Model IV shows that both VD3 insufficiency and sufficiency were associated with a reduced likelihood of cognitive impairment compared to VD3 deficiency among participants with fair/poor sleep quality. On the other hand, among participants with good sleep quality, only VD3 sufficiency was marginally associated with a lower likelihood of cognitive impairment compared to VD3 deficiency.

Model V provides further evidence that individuals who experienced VD3 deficiency and had a shorter sleep duration were more likely to exhibit cognitive impairment than those who were VD3 deficient but had a normal sleep duration. However, for participants with normal or longer sleep durations, having sufficient levels of VD3 was only marginally associated with lower odds of cognitive impairment compared to participants with normal sleep duration but VD3 deficiency. Overall, the longitudinal interaction analyses reveal some significant longitudinal associations between VD3 and cognitive impairment and some weak associations with cognitive impairment for the interaction between the level of VD3 and sleep quality/duration.

## 4. Discussion

This study is a unique exploration of the associations between serum levels of VD3 concentration and sleep quality/duration in relation to cognitive impairment among older adults from eight blue zones in China. The present study found that poor sleep quality and long sleep duration (≥9 h) were associated with an increased risk of cognitive impairment than good sleep quality and normal sleep duration (7–8 h), respectively, although the relationship between the sleep duration and cognitive functioning was explained by baseline health conditions. The positive role of sleep quality against cognitive impairment is generally consistent with past research. For instance, studies in both Canada and the U.S. have shown that poor sleep quality is significantly related to subsequent cognitive decline among older adults [28]. A study in Guangdong Province, China, also found that lower sleep quality was associated with higher prevalence of cognitive impairment [61]. As for sleep durations, several meta-analyses suggested that longer sleep duration may elevate the risk of cognitive impairment among older adults [24,62]. Moreover, our results are also in line with some previous studies that the significant relationship between long sleep durations and cognitive function could be substantially attenuated by health status [63].

Importantly, our research further contributes to the existing literature by demonstrating that sufficient VD3 levels combined with good sleep and normal sleep duration (7–8 h) could have an enhanced positive association with cognitive function among older Chinese; by contrast, the deficient VD3 levels could make poor cognitive performance worsen if it was coupled with fair/poor sleep quality and short or long sleep duration. It is widely recognized that low VD3 concentration is consistently associated with cognitive impairment and various neurodegenerative diseases, including all-cause dementia in older adults and animal studies [33,34,35,36,37,64,65]. Our findings partially corroborate the findings from a study conducted by Song and Wu [66] who found an increased excessive risk of cognitive impairment in older adults in China with heart failure who reported poor sleep quality and had low levels of VD3 concentration compared to those who reported either poor sleep quality or had a low level of VD3 concentration.

Several potential mechanisms may explain the observed association. First, sleep is a critical physiological process that restores functional capacity and maintains homeostasis [67]. Evidence indicates that long sleep duration may reflect circadian disruptions related to sleep dysregulation and impaired cognition [27]. Meanwhile, elevated levels of interleukin-6 (IL-6) and C-reactive protein (CRP) have been found in long-hour sleepers, suggesting a possible association between long sleep duration, increased inflammation, and impaired cognition [67].

Second, several studies found that lower levels of VD3 concentration were evidenced to be associated with increased odds of cognitive impairment and dementia among older adults [31,33,34,35,36], suggesting that VD3 may play a critical role in the etiology of cognitive impairment, Alzheimer’s disease, and all-cause dementia. VD3 deficiency may increase the risk of many chronic illnesses, such as stroke, diabetes, and hypertension, which may in turn give rise to cognitive impairment [68]. Numerous animal studies also demonstrate that the level of VD3 concentration affects antioxidative mechanisms, neuronal calcium homeostasis, and the expression of neurotrophic factors that are closely linked with cognitive function [35,69,70], and that high levels of VD3 concentration could enhance detoxification and eventually maintain cognitive function [71,72].

Third, it is reasonable to assume that maintaining an adequate level of concentration of VD3 among older adults with good sleep quality may offer enhanced protection against cognitive decline, and the co-existence of a poor sleep pattern and VD3 deficiency may lead to a worsened risk of cognitive impairment [9,10,17,25,29,64]. Furthermore, as reviewed earlier, the increased level of VD3 concentration could improve sleep quality [43,44]. Therefore, preserving sufficient levels of VD3 concentration can also be justified as a means to mitigate the negative impact of poor sleep patterns on cognitive function [73,74]. We strongly encourage future research endeavors aimed at validating our findings and unraveling the underlying mechanisms that link VD3 levels, sleep quality and duration, and cognitive impairment in older adults.

However, we also found that the longitudinal associations between sleep quality/duration and subsequent cognitive impairment were very weak, and so did the interactions between the level of VD3 and sleep quality/duration. This could be attributed to several factors. First, the impact of sleep on cognitive function is likely to accumulate over time or occur simultaneously with other factors, making it challenging to detect short-term significant longitudinal associations between sleep quality/duration and cognitive impairment. Second, the validity of measuring overall sleep quality and duration over a period of three years could be compromised by the common occurrence of short-term changes in sleep quality/duration [75,76]. Future research could benefit from collecting data on more complete episodes of sleep quality/duration and cognitive performance throughout the survey interval.

One unique aspect of the current research is the inclusion of a large proportion of the oldest-old subjects in the sample. Previous studies, as indicated by several meta-analyses [24,43,44,59,62], have rarely included older adults in their sample, not to mention the oldest-old adults. To the best of our knowledge, our research is the first to examine the interactions between VD3, sleep quality/duration, and their relationship with cognitive function among a sample primarily composed of oldest-old adults. Overall, our findings have implications for the maintenance of cognitive functioning in older adults, which is a crucial aspect of healthy aging, particularly regarding adequate VD3 intake, good sleep quality, and sufficient sleep duration.

Several limitations should be taken into account when interpreting the findings of this study. First, information on the sleep quality and duration in HABCS was self-reported, which may suffer from recall biases. Nevertheless, such biases should not be substantial because self-reported sleep duration was well adapted to sleep durations based on polysomnography and actigraphy [77]. Self-reported questions also have some merits, including simplicity, ease of understanding, and the ability to provide a summary or an overall measure for a status/behavior that the detailed scale or a series of questions may not be able to capture. Considering that respondents in this study are relatively old (mean age is 86.5 years) and less educated (nearly two-thirds had no education; only less than 9% received 7+ years of schooling), the self-reported measures have some merits. Second, daytime napping was not asked explicitly in HABCS, which prohibits us from differentiating it from the nocturnal sleep amount. However, it may not be a severe issue because empirical evidence has suggested that daytime napping and nocturnal sleep may share similar mechanisms with cognitive functioning [78]. Third, it should be noted that the samples used in this study were collected from only eight specific blue zones in China. Therefore, the results in the present study may not necessarily be applicable to the whole population of China. Additionally, it is worth mentioning that the sample weight was not provided in this study, which would have allowed for a more accurate representation of the population in these eight areas. Fortunately, we controlled key demographics, disease conditions, and disabilities that are closely linked with cognitive function, and such biases would not be substantial [79,80,81]. Nevertheless, further research is needed to address these limitations and ensure more reliable and comprehensive findings.

## 5. Conclusions

Using a sample taken from eight blue zones in China, our study found that individuals with poor sleep quality and long sleep duration (≥9 h) had a higher likelihood of cognitive impairment, even when considering various potential confounding factors. However, such significant relationships were mainly concurrent or cross-sectional, rather than longitudinal. Furthermore, our sleep quality/duration and VD3 interaction analyses revealed a significant protective effect of sufficient VD3 concentration against cognitive impairment among the study sample. Conversely, inadequate levels of VD3, coupled with poor sleep quality or either insufficient or excessive sleep durations, can exacerbate cognitive impairment. However, further research is granted to gain a deeper understanding of the potential mechanisms underlying the complicated connections between vitamin D3 levels, sleep quality, sleep duration, and cognitive decline in older adults.

## Figures and Tables

**Table 1:** Sample distribution by study variables, Healthy Aging and Biomarkers Cohort Study (HABCS), 2011 and 2014.

	All Samples	Level of VD3		
		Deficient (VD3 < 25 nmol/L)	Insufficient (VD3 25–50 nmol/L)	Sufficient (VD3 ≥ 50 nmol/L)
**Characteristics**				
**Total sample size (#n)**	4816	987	2382	1447
share of the total sample	100.0	20.5	49.5	30.0
**Mean VD3 (SD)**	42.6 (22.2)	18.1 (5.1)	36.5 (7.0)	69.3 (18.9)
**Cognitive health**				
Mean MMSE score (SD)	22.8 (9.5)	17.4 (11.23)	23.4 (8.99)	25.6 (7.18)
Cognitively impaired	31.7	55.82	29.8	18.4
**Sleep**				
Good sleep quality	63.0	65.9	65.4	57.0
≤6 h of sleep	31.1	25.6	30.0	36.8
7–8 h of sleep	44.1	41.5	47.1	41.0
≥9 h of sleep	24.7	32.8	22.9	22.2
**Demographic variables**				
Mean age in years (SD)	86.5 (11.76)	92.5 (10.86)	86.1 (11.38)	83.1 (11.41)
Aged 80 or older	69.2	84.9	68.9	59.0
Men	44.2	23.0	42.6	61.4
Urban	19.4	23.4	22.2	11.9
Han ethnicity	91.2	92.3	90.1	92.1
**Socioeconomic status (SES)**				
0 years of schooling	63.7	78.2	64.1	53.3
1–6 years of schooling	27.4	17.1	27.4	34.0
7+ years of schooling	8.9	4.7	8.5	12.7
Economically independent	24.2	14.0	24.3	31.0
Professional job	3.7	1.8	4.0	4.4
Economically sufficient	85.8	87.8	86.3	83.8
Economically rich	15.5	17.5	16.4	12.5
Adequate access to healthcare	95.9	95.7	95.9	96.1
**Family/Social connections**				
Currently married	38.1	21.2	38.0	49.6
Living with family member(s)	75.6	80.1	76.2	71.7
**Health practices**				
Smoking at present	15.4	8.7	14.5	21.3
Drinking at present	14.5	9.1	14.0	19.0
Doing regular exercise	15.4	9.4	15.4	19.4
**Health condition**				
Activities of daily living (ADL) disabled	22.0	48.1	18.9	9.3
Instrumental ADL (IADL) disabled	62.2	83.4	59.9	51.3
Having 1+ chronic disease	55.2	52.4	55.8	55.8
Poor self-rated health (SRH)	12.7	16.1	12.1	11.3
**Wave**				
The 2014 wave	51.1	52.8	52.1	48.3
**The eight areas**				
Rudong, Jiangsu Province	13.9	15.0	16.4	9.1
Leizhou, Shandong Province	17.6	30.7	17.5	8.7
Xiayi, Henan Province	21.9	22.0	19.0	26.5
Zhongxiang, Hubei Province	10.6	11.1	13.9	4.8
Mayang, Hunan Province	7.7	7.3	8.1	7.1
Foshan, Guangdong Province	9.3	8.1	8.8	10.9
Yongfu, Guangxi Province	6.3	4.2	7.7	5.2
Chengmai, Hainan Province	12.9	1.5	8.7	27.7

Note: (1) All results are unweighted, based on the pooled dataset of the 2011 and 2014 waves with 4816 observations. The outcome of the 3385 participants is similar. (2) Mini-mental State Examination (MMSE) scores and age were measured in mean, whereas all other variables are measured in percentages, and the numbers in the parentheses are standard deviation (SD). (3) The sum of percentages may not equal 1 due to rounding. (4) VD3: Serum 25-hydroxyvitamin D_3_ (25(OH)D_3_).

**Table 2:** Odds ratios of cognitive impairment for the levels of VD3 concentration and sleep quality and duration, the pooled dataset of the 2011 and 2014 waves of the Healthy Aging and Biomarkers Cohort Study (HABCS).

	Model I	Model II	Model III	Model IV	Model V
**Level of VD3**					
*Deficient (reference)*	1.00	1.00	1.00		
Insufficient	0.57 ***	0.60 ***	0.83		
Sufficient	0.37 ***	0.42 ***	0.67 *		
**Sleep quality**					
*Fair/poor (reference)*	1.00	1.00	1.00		1.00
Good (fair/poor)	0.49 ***	0.53 ***	0.66 **		0.53 *
Sleep hours					
≤6 h	0.84	0.87	0.90	0.90	
*7–8 h (reference)*	1.00	1.00	1.00	1.00	
≥9 h	1.30 *	1.30 *	1.16	1.15	
**Interactions between VD3 and sleep quality**					
*Fair/poor quality and VD3 deficient (reference)*				1.00	
Fair/poor but VD3 insufficient				1.16	
Fair/poor but VD3 sufficient				0.99	
Good but VD3 deficient				0.99	
Good but VD3 insufficient				0.70 ^+^	
Good and VD3 sufficient				0.53 **	
**Interactions between VD3 and sleep hours**					
≤6 h and VD3 deficient					0.68
≤6 h and VD3 insufficient					0.48 **
≤6 h and VD3 sufficient					0.36 ***
*7–8 h and VD3 deficient (reference)*					1.00
7–8 h and VD3 insufficient					0.54 **
7–8 h and VD3 sufficient					0.35 ***
≥9 h and VD3 deficient					1.17
≥9 h and VD3 insufficient					0.71 ^+^
≥9 h and VD3 sufficient					0.51 *
−Log likelihood	2026.3	1970.7	1832.9	1830.4	1969.9
rho	0.37 ***	0.35 ***	0.29 ***	0.29 ***	0.35 ***
df	19	29	33	35	33
Sample size (N)	4816	4816	4816	4816	4816

Note: (1) Odds ratios were obtained from multilevel random intercept models. (2) Model I adjusted demographic variables, years of schooling, provinces, and the year of survey waves; Model II further adjusted economic status, primary occupation, and access to medical services; family and social connections; and health practices; and Model III additionally adjusted health conditions. Model IV and Model V examined the interactions between VD3 and sleep quality and between VD3 and sleep duration, respectively, controlling for all covariates in Model III. (3) The outcome variable of cognitive impairment was defined when a Mini-Mental State Examination score is less than 24. (4) VD3 deficient means 25(OH)D ≤ 25 nmol/L (10 mg/L), insufficient means 25 nmol/L (10 mg/L) < 25(OH)D ≤ 50 nmol/L (10 mg/L), and sufficient means 25(OH)D > 50 nmol/L (10 mg/L). (5) The category in italics is the reference group of a given variable. (6) ^+^ *p* < 0.1; * *p* < 0.05; ** *p* < 0.01; *** *p* < 0.001.

**Table 3:** Odds ratios of cognitive impairment for the levels of VD3 concentration and sleep quality/duration, longitudinal analysis based on the panel dataset from 2011 to 2014, the Healthy Aging and Biomarkers Cohort Study (HABCS).

	Model I	Model II	Model III	Model IV	Model V
**Level of VD3**					
*Deficient (reference)*	1.00	1.00	1.00		
Insufficient	0.62 ***	0.59 *	0.63 *		
Sufficient	0.41 ***	0.39 ***	0.45 **		
**Sleep quality**					
*Fair/poor (reference)*	1.00	1.00	1.00		
Good	1.07	1.17	1.25		
**Sleep hours**					
≤6 h	1.32	1.35	1.39	1.38	
*7–8 h (reference)*	1.00	1.00	1.00	1.00	
≥9 h	1.01	0.98	0.98	0.97	
**Interactions between VD3 and sleep quality**					
*Fair/poor quality and VD3 deficient (reference)*				1.00	
Fair/poor but VD3 insufficient				0.53 ^+^	
Fair/poor but VD3 sufficient				0.46 *	
Good but VD3 deficient				1.09	
Good but VD3 insufficient				0.77	
Good and VD3 sufficient				0.47 ^+^	
**Interactions between VD3 and sleep hours**					
≤6 h and VD3 deficient					2.65 *
≤6 h and VD3 insufficient					0.87
≤6 h and VD3 sufficient					0.63
*7–8 h and VD3 deficient (reference)*					1.00
7–8 h and VD3 insufficient					0.75
7–8 h and VD3 sufficient					0.52 ^+^
≥9 h and VD3 deficient					1.08
≥9 h and VD3 insufficient					0.77
≥9 h and VD3 sufficient					0.40 ^+^
−Log likelihood	549.1	534.5	525.0	524.4	532.9
df	18	28	32	34	32
Sample size (N)	1404	1404	1404	1404	1404

Note: (1) Odds ratios were obtained from longitudinal logit regression models. (2) Model I adjusted demographic variables, years of schooling, provinces, and the year of survey waves; Model II further adjusted economic status, primary occupation, and access to medical services; family and social connections; and health practices; and Model III additionally adjusted health conditions. Model IV and Model V examined the interactions between VD3 and sleep quality and between VD3 and sleep duration, respectively, controlling for all covariates in Model III. (3) The outcome variable cognitive impairment was defined when a Mini-Mental State Examination score is less than 24. (4) VD3 deficient means 25(OH)D ≤ 25 nmol/L (10 mg/L), insufficient means 25 nmol/L (10 mg/L) < 25(OH)D ≤ 50 nmol/L (10 mg/L), and sufficient means 25(OH)D > 50 nmol/L (10 mg/L). (5) The category in italics is the reference group of a given variable. (6) ^+^ *p* < 0.1; * *p* < 0.05; ** *p* < 0.01; *** *p* < 0.001.

## Data Availability

The dataset is publicly available at the National Archive of Computerized Data on Aging, University of Michigan (https://www.icpsr.umich.edu/web/NACDA/studies/37226, accessed on 25 September 2023). We obtained the data from this portal. The data are also publicly available via the link: https://opendata.pku.edu.cn/dataverse/CHADS;jsessionid=1cdca5925c8cc2b67b83a5d4b9bc (accessed on 25 September 2023).

## Supplementary Materials

The following supporting information can be downloaded at: https://www.mdpi.com/article/10.3390/nu15194192/s1, Table S1. Research design by model; Table S2. Linear coefficients of cognitive impairment for the levels of VD3 concentration, sleep quality/duration, the pooled dataset of the 2011 and 2014 waves of the Healthy Aging and Biomarkers Cohort Study (HABCS); Table S3. Linear coefficients of cognitive impairment for the levels of VD3 concentration, sleep quality/duration, the panel dataset from the 2011 to 2014 wave of the Healthy Aging and Biomarkers Cohort Study (HABCS).
