# Supplementary material for: Serum Vitamin D3 Concentration, Sleep, and Cognitive Impairment among Older Adults in China
Source: Nutrients. 2023 Sep 28;15(19):4192. doi: 10.3390/nu15194192 (PMC10574235; doi:10.3390/nu15194192)
Supplement: Supplementary file 1 [file nutrients-15-04192-s001.zip › nutrients-2593765-supplementary.pdf]

Supplementary Materials

Table S1. Research design by model.

	Model I	Model II	Model III	Model IV	Model V
provinces and the year of survey waves	√	√	√	√	√
(a) demographic characteristics	√	√	√	√	√
(b_1) socioeconomic status (i.e., education)	√	√	√	√	√
(b_2) socioeconomic status (variables other than education)		√	√	√	√
(c) family and social connections		√	√	√	√
(d) health practices		√	√	√	√
(e) health condition			√	√	√
interactions (VD3 and sleep quality)				√	
interactions (VD3 and sleep duration)					√

Note: Education was measured by years of schooling. VD3 refers to the level of serum 25-hydroxyvitamin D_3 ($25(OH)D_3$).

Table S2. Linear coefficients of cognitive impairment for the levels of VD3 concentration, sleep quality/duration, the pooled dataset of the 2011 and 2014 waves of the Healthy Aging and Biomarkers Cohort Study (HABCS).

	**Model I**	**Model II**	**Model III**	**Model IV**	**Model V**
**VD3**					
Deficient (reference)	0.00	0.00	0.00		
Insufficient	2.13***	1.98***	0.98***		
Sufficient	2.68***	2.42***	1.18***		
**Sleep quality**					
Fair/poor (reference)	0.00	0.00	0.00		0.00
Good	1.40***	1.28***	0.85***		1.29***
**Sleep hours**					
≤ 6 hours	0.42	0.42	0.29	0.28	
7-8 hours (reference)	0.00	0.00	0.00	0.00	
≥ 9 hours	- 0.82**	- 0.83**	- 0.51+	- 0.51+	
**Interactions between VD3 and sleep quality**					
fair/poor quality and VD3 deficient(reference)					
fair/poor but VD3 insufficient				0.44	
fair/poor but VD3 sufficient				0.81	
good but VD3 deficient				0.27	
good but VD3 insufficient				1.53***	
good and VD3 sufficient				1.64***	
**Interactions between VD3 and sleep hours**					
≤ 6 hours and VD3 deficient					0.65
≤ 6 hours and VD3 insufficient					2.06***
≤ 6 hours and VD3 sufficient					2.57***
7-8 hours and VD3 deficient (reference)					
7-8 hours and VD3 insufficient					1.79***
7-8 hours and VD3 sufficient					1.97***
≥ 9 hours and VD3 deficient					- 1.81***
≥ 9 hours and VD3 insufficient					0.99*
≥ 9 hours and VD3 sufficient					1.95***
R-squared	0.35	0.37	0.44	0.44	0.37
rho	0.50***	0.49***	0.44***	0.44***	0.48***
df	19	29	33	35	33
Sample size (N)	4816	4816	4816	4816	4816

Note: (1) The linear coefficients were obtained from multilevel random intercept models from the 2011 and 2014 pooled dataset. The value of rho indicates the intrapersonal correlation between waves. (2) Model I only adjusted for demographic variables, years of schooling, provinces, and the year of survey waves; Model II further adjusted economic status, primary occupation, and access to medical services, family/social connections and health practices; and Model III additionally adjusted health conditions. Model IV and Model V reported the results on the interactions between VD3 and sleep quality and between VD3 and sleep duration, respectively, controlling for all covariates in Model III. (3) The outcome measured by the Mini-Mental State Examination scores (ranging 0-30). (4) VD3 deficient means 25(OH)D ≤ 25 nmol/L (10 mg/L), insufficient means 25 nmol/L (10 mg/L) < 25(OH)D ≤ 50 nmol/L (10 mg/L) and sufficient means 25(OH)D > 50 nmol/L (10 mg/L). (5) The category in italic is the reference group of a given variable. (6) +p<0.1; *p<0.05; **p<0.01; ***p<0.001.

Table S3. Linear coefficients of cognitive impairment for the levels of VD3 concentration, sleep quality/duration, the panel dataset from the 2011 to 2014 wave of the Healthy Aging and Biomarkers Cohort Study (HABCS).

	**Model I**	**Model II**	**Model III**	**Model IV**	**Model V**
**VD3**					
Deficient (reference)	0.00	0.00	0.00		
Insufficient	2.35***	2.33***	1.76**		
Sufficient	2.82***	2.81***	2.06**		
**Sleep quality**					
Fair/poor (reference)	1.00	0.00	0.00		
Good	0.02	- 0.15	- 0.35		- 0.15
**Sleep hours**					
≤ 6 hours	- 0.14	- 0.19	- 0.30	- 0.28	
7-8 hours (reference)	0.00	0.00	0.00	0.00	
≥ 9 hours	- 0.97+	- 0.90+	- 0.53	- 0.53	
**Interactions between VD3 and sleep quality**					
fair/poor quality and VD3 deficient(reference)					
fair/poor but VD3 insufficient				1.43	
fair/poor but VD3 sufficient				1.54	
good but VD3 deficient				- 0.88	
good but VD3 insufficient				1.06	
good and VD3 sufficient				1.49	
**Interactions between VD3 and sleep hours**					
≤ 6 hours and VD3 deficient					- 1.41
≤ 6 hours and VD3 insufficient					1.61+
≤ 6 hours and VD3 sufficient					2.27*
7-8 hours and VD3 deficient (reference)					
7-8 hours and VD3 insufficient					1.89*
7-8 hours and VD3 sufficient					1.75+
≥ 9 hours and VD3 deficient					- 1.80
≥ 9 hours and VD3 insufficient					0.48
≥ 9 hours and VD3 sufficient					2.30*
Adj R-squared	0.37	0.38	0.40	0.40	0.38
df	18	28	32	34	32
Sample size (N)	1404	1404	1404	1404	1404

Note: (1) The linear coefficients were obtained from longitudinal linear models from the 2011 and 2014 panel dataset. (2) Model I only adjusted for demographic variables, years of schooling, provinces, and the year of survey waves; Model II further adjusted economic status, primary occupation, and access to medical services, family/social connections and health practices; and Model III additionally adjusted health conditions. Model IV and Model V reported the results on the interactions between VD3 and sleep quality and between VD3 and sleep duration, respectively, controlling for all covariates in Model III. (3) The outcome measured by the Mini-Mental State Examination scores (ranging 0-30). (4) VD3 deficient means 25(OH)D ≤ 25 nmol/L (10 mg/L), insufficient means 25 nmol/L (10 mg/L) < 25(OH)D ≤ 50 nmol/L (10 mg/L) and sufficient means 25(OH)D > 50 nmol/L (10 mg/L). (5) The category in italic is the reference group of a given variable. (6) +p<0.1; *p<0.05; **p<0.01; ***p<0.001.
